# Spectrum and prognosis of CAR-T-related endocrine toxicity: glucose/calcium dysregulation, pituitary axis disorders, and overlap with CRS

**DOI:** 10.3389/fmed.2026.1786129

**Published:** 2026-04-14

**Authors:** Jizhou Liang, Fangyuan Hu, Jiaxun Li, Yinghong Zhai, Xiaojing Guo, Jinfang Xu, Xiaofei Ye, Jia He

**Affiliations:** 1 Faculty of Military Health Service, Naval Medical University, Shanghai, China; 2 Clinical Research Unit, School of Medicine, Shanghai Ninth People’s Hospital Affiliated to Shanghai JiaoTong University, Shanghai, China

**Keywords:** CAR T-cell therapy, cytokine release syndrome, drug safety, endocrine adverse events, FAERS, pharmacovigilance

## Abstract

**Background:**

Chimeric antigen receptor (CAR) T-cell therapy is increasingly used for hematologic malignancies, yet the spectrum and clinical significance of its endocrine adverse events (AEs) remain poorly characterized.

**Objective:**

To identify and clinically contextualize signals of endocrine AEs associated with CAR T-cell therapy, with a focus on their overlap with cytokine release syndrome (CRS).

**Methods:**

We performed a retrospective disproportionality analysis using the FDA Adverse Event Reporting System (FAERS) database (2017 to Q2 2025). Reporting odds ratio (ROR) and information component (IC) were calculated for endocrine AEs associated with six CAR T-cell products. To validate and elaborate these pharmacovigilance signals, a structured literature review was conducted to identify published clinical evidence.

**Results:**

The FAERS analysis identified 269 endocrine AE reports, yielding 14 significant disproportionality signals. Hyperglycemia was the most frequently reported event, while estrogen deficiency showed the strongest signal strength (ROR₀₂₅ = 14.93). Signals for adrenal insufficiency and hypothalamo-pituitary disorders were primarily associated with axicabtagene. Critically, mortality was frequently reported among cases with positive endocrine signals, particularly when the endocrine AE co-occurred with CRS. The literature review provided direct clinical validation: a retrospective study confirmed a 39% incidence of CRS-associated hyperglycemia, and independent case reports documented the first instances of CAR T-cell therapy-induced Hashimoto’s thyroiditis and central diabetes insipidus. These clinical cases corroborated the FAERS signals and suggested immune-inflammatory mechanisms, often independent of corticosteroid use.

**Conclusion:**

This integrated analysis reveals a distinct spectrum of CAR T-cell-related endocrine toxicities, encompassing glucose and calcium dysregulation, pituitary axis disorders, and autoimmune phenomena. The frequent and potentially fatal overlap with CRS underscores the need for enhanced clinical vigilance. Proactive monitoring of endocrine function, especially in patients experiencing CRS, is warranted to mitigate these underrecognized complications.

## 1 Introduction

Chimeric antigen receptor (CAR) T-cell therapy is a breakthrough method of immunotherapy and a novel approach to cancer treatment. This method involves the genetic engineering of a patient’s T cells to express receptors that are particularly sensitive to antigens found on the tumor surface. Consequently, these modified T cells are able to target and eliminate tumor cells effectively upon reinfusion into the patient’s body (1, 2). This therapy has shown great promise in treating particular forms of cancer, particularly hematological malignancies (3).

Eleven CAR T-cell therapy products have been approved and marketed globally. Among these are Novartis’ CD19-targeted tisagenlecleucel (Kymriah®) (4), axicabtagene ciloleucel (Yescarta®) and brexucabtagene autoleucel (Tecartus®), which were developed by Kite Pharma (5–7). In 2021, Bristol-Myers Squibb’s CART-cell products, Breyanzi and Abecma, received marketing approval in the United States. Additionally, in 2022, Legendary Biologics obtained marketing approval for its CAR T-cell product, Carvykti (ciltacabtagene autoleucel) (8).

However, with the wide use of CAR T-cell therapy, questions about its possible harm have increased (9, 10). A range of adverse effects (11–13), including neurotoxicity (14), cardiotoxicity (15) and ocular toxicity (16), have been observed in patients treated with CAR T-cell products. Among the most common adverse events (AEs) linked to CAR T-cell therapy are cytokine release syndrome and immune effector cell-associated neurotoxicity syndrome (ICANS) (10).

Despite the rapidly expanding clinical use of CAR-T therapies, published evidence characterizing endocrine adverse events remains sparse. Beyond the well-documented CRS and ICANS, isolated case reports have described metabolic complications such as hyperglycemia during treatment, as well as rare endocrine manifestations including Hashimoto’s thyroiditis and central diabetes insipidus. However, no systematic evaluation of endocrine safety signals has been conducted. This evidence gap limits the interpretation of pharmacovigilance data and hinders the development of evidence-based monitoring protocols. Thus, this study aimed to identify endocrine adverse event signals associated with CAR-T cell therapy using the FDA Adverse Event Reporting System (FAERS) and to contextualize these signals within the existing clinical and mechanistic literature.

## 2 Methods

### 2.1 Disproportionality analysis setting

Covering the period from 2017 to the second quarter of 2025, the FAERS database provided the data for this real-world retroactive study. The FAERS database is a worldwide spontaneous reporting system (SRS) computed quarterly by the United States. Compared with the more than 20 million adverse event (AE) records recorded by doctors, patients, and healthcare organizations from more than 100 countries, the FDA is among the largest publicly available pharmacovigilance databases in the globe. Regulatory authorities have completely anonymized the database (17, 18).

The FAERS database records patient adverse reaction occurrence under the “preferred term” (PT) level of medical terminology derived from the Medical Dictionary for Regulatory Activities (MedDRA 27.0).

We have included all the CAR-T therapies that have currently been approved by the FDA in the study: axicabtagene ciloleucel (Yescarta), tisagenlecleucel (Kymriah), idecabtagene vicleucel (Abecma), brexucabtagene autoleucel (Tecartus), ciltacabtagene autoleucel (Carvykti), and lisocabtagene maraleucel (Breyanzi).

### 2.2 Data processing and analysis

Seven tables totaling many variables make up the FAERS database. Before the data analysis started, duplicates were eliminated via a variable matching approach after basic variables were taken from the data files. During the variable matching process, records having the same key variables—e.g., report ID, basic patient information, suspected drug, etc.—were regarded as duplicate reports. The most recent report was kept in cases of several instances of the same record, while the others were deleted. When used as screening variables for CAR T-cell-related records in the present study, the drug description in the report included both the brand name and the generic name. Seven categories of adverse reaction outcomes are defined: death, life-threatening events, hospitalization, disability, congenital anomalies, the need for intervention to prevent permanent damage or impairment, and other major medical conditions. Moreover, one of four categories—PS (primary suspect), SS (secondary suspect), C (concomitant), and I (interaction)—was assigned to each drug found in every adverse reaction report. To guarantee the strength of the signal, only records linked to CAR T-cell treatments categorized as either “primary suspect” or “secondary suspect” were chosen for study.

Disproportionality analysis of the data helped to enable a comparison between the target adverse reactions and those of other drugs as well as between the proportionality of the target drug and that of other drugs. This method was used in search of possible safety concerns related to the drugs in question. Bayesian confidence propagation neural networks of information component (IC) and proportional reports reporting odds ratio (ROR) were used to investigate the disproportionality between drugs and adverse reactions (19, 20). A situation in which a given adverse reaction is linked to a specific drug is said to be “disproportionality” on the basis of information component values (ICs) and elevated reporting odds ratios (RORs). When the lower limit of the 95% confidence interval of the ROR (ROR025) estimate for a particular drug-adverse reaction combination was greater than 1 or when the lower limit of the IC (IC025) value was greater than 0, and provided that at least three reports were available, a statistically significant association was deemed to exist (18).

SAS 9.4 software (SAS Institute, NC, United States) was used in this study to process complex data and perform comprehensive statistical evaluations. The identification of signals in this work does not suggest the development of a causal relationship. These signals should be taken as first hints of possible problems or events deserving of more research. By means of further investigation, it is imperative to validate and build upon these initial observations to improve the validity of these conclusions and support developments in clinical practice and drug safety control.

### 2.3 Literature search for evidence contextualization

To contextualize the pharmacovigilance signals identified from FAERS, we conducted a structured and systematic literature search. The objective was to map existing clinical and mechanistic evidence relevant to CAR-T-associated endocrine adverse events, rather than to perform a formal systematic review or meta-analysis with quantitative synthesis.

We searched electronic databases including PubMed, Embase, Web of Science, and the Cochrane Library from inception to December 2025. Additional sources included ClinicalTrials.gov, FDA product labels, and relevant clinical guidelines. The search strategy combined terms for CAR-T products (“chimeric antigen receptor,” “CAR-T,” “axicabtagene,” “tisagenlecleucel,” “idecabtagene,” “brexucabtagene,” “ciltacabtagene,” “lisocabtagene”) with endocrine-related terms (“endocrine,” “hyperglycemia,” “hypercalcemia,” “adrenal insufficiency,” “pituitary,” “diabetes insipidus,” “thyroid,” “thyroiditis,” “hypogonadism,” “estrogen deficiency”). Studies were eligible if they included patients receiving any FDA-approved CAR-T product and reported endocrine or metabolic outcomes. All study designs were considered, including clinical trials, retrospective studies, case reports, and mechanistic investigations. Publications on immune checkpoint inhibitor-associated endocrinopathies were reviewed as analogous evidence to support biological plausibility where direct CAR-T data were limited.

Two reviewers independently screened titles, abstracts, and full texts. The literature search and selection process followed the PRISMA 2020 framework for transparency. Data extracted included study design, patient population, CAR-T product, adverse event type, timing, severity, and proposed mechanisms. Given the limited number of directly relevant studies, formal quantitative synthesis was not attempted. Instead, findings were narratively synthesized and integrated with the FAERS disproportionality results to support evidence interpretation.

## 3 Results

### 3.1 Disproportional analysis

#### 3.1.1 Descriptive analysis

**Figure 1:**
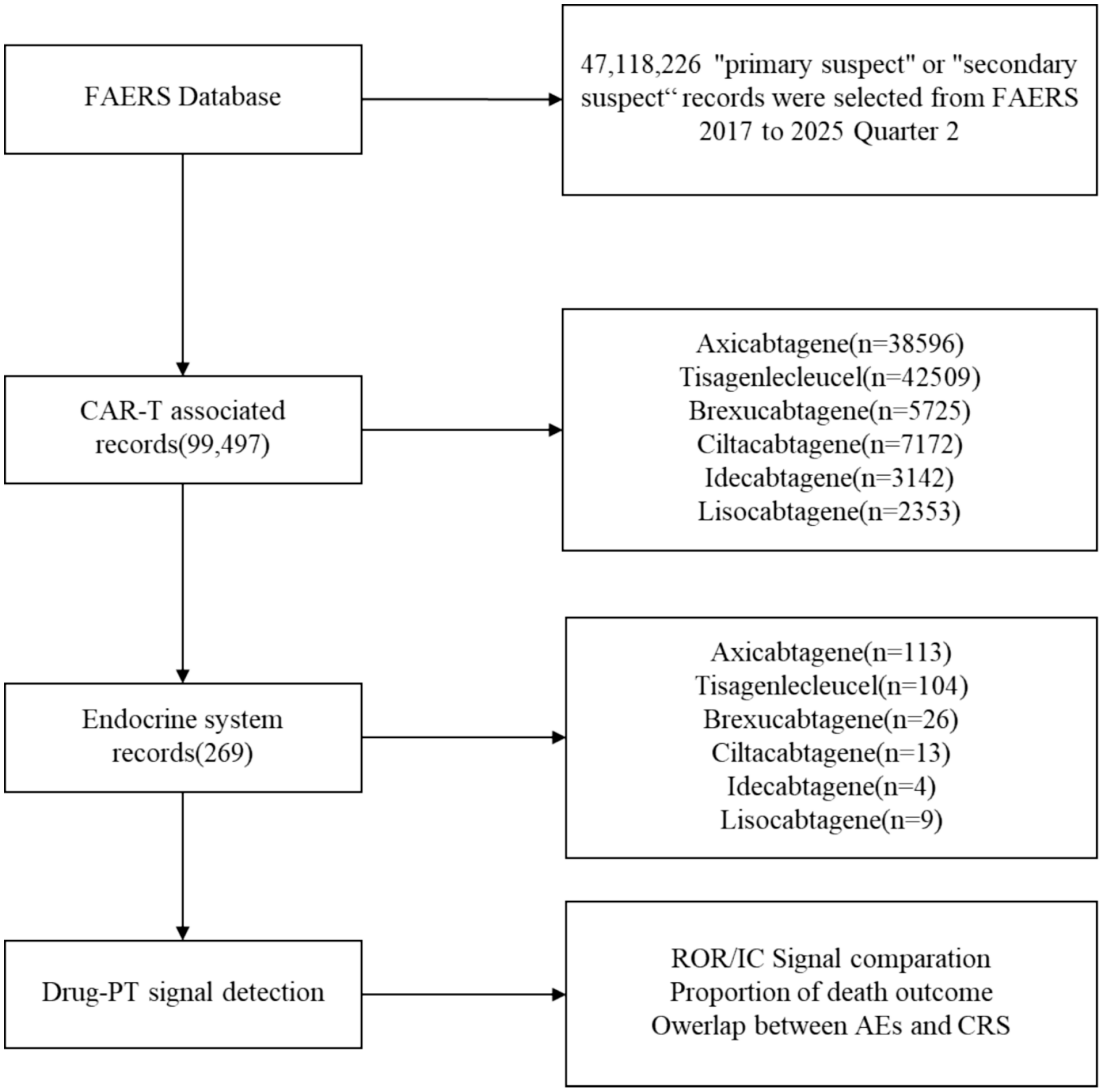
Flow chart about the process of selecting records from the FAERS database. Over the course of the study, which spanned from 1 January 2017 to 30 June 2025, a total of 47,118,226 primary suspected records were selected. A total of 99,497 records were identified as being associated with CAR-T, while 269 records were considered to potentially relate to the endocrine system. The calculation of disproportionality was performed for drug event combinations comprising a minimum of three records. CAR-T, Chimeric antigen receptor T cell; FAERS, US FDA Adverse Event Reporting System; PT, Preferred term.

The FAERS database had 47,118,226 records available overall during the study interval. Of these, 99,497 records were investigated for CAR T-cell therapy either as a main or secondary suspected agent. As shown in Figure 1, this screening identified 113 cases of endocrine system-related AEs linked with axicabtagene, 104 cases with tisagenlecleucel, 26 cases with brexucabtagene, 13 cases with ciltacabtagene, 4 cases with idecabtagene, and 9 cases with lisocabtagene. Table 1 shows the pertinent demographic data together with the clinical results.

**Table 1:** CAR-T therapy design and baseline characteristics of reported records with CAR-T-associated endocrine adverse events.

Category	Item	Axicabtagene (*n* = 113)	Brexucabtagene (*n* = 26)	Ciltacabtagene (*n* = 13)	Idecabtagene (*n* = 4)	Lisocabtagene (*n* = 9)	Tisagenlecleucel (*n* = 104)	CAR-T (*n* = 269)
Design	Antigen target	CD19	CD19	BCMA	BCMA	CD19	CD19	-
	Co-stimulatory domain	CD28	CD28	4-1BB	4-1BB	4-1BB	4-1BB	-
Baseline characteristics	Age median (IQR)	57 (47–65)	61 (56–66)	68 (19–79)	67 (63–69.5)	70 (65–74)	20.5 (15–67.5)	58.5 (26–67)
	Weight median (IQR)	83.90 (66.45–96.70)	105.70 (69.40–108.30)	55.90 (54.30–66.00)	135.50 (106.00–165.00)	88.75 (77.60–117.50)	67.10 (38.00–82.30)	77.75 (60.00–91.00)
	Gender (%)							
	Female	48 (42.48)	2 (7.69)	5 (38.46)	1 (25.00)	2 (22.22)	47 (45.19)	105 (39.03)
	Male	57 (50.44)	19 (73.08)	6 (46.15)	3 (75.00)	7 (77.78)	49 (47.12)	141 (52.42)
	Unknown	8 (7.08)	5 (19.23)	2 (15.38)	0 (0.00)	0 (0.00)	8 (7.69)	23 (8.55)
	Event year (%)							
	2018	7 (6.19)	0 (0.00)	0 (0.00)	0 (0.00)	0 (0.00)	5 (4.81)	12 (4.46)
	2019	15 (13.27)	0 (0.00)	0 (0.00)	0 (0.00)	0 (0.00)	27 (25.96)	42 (15.61)
	2020	22 (19.47)	0 (0.00)	0 (0.00)	0 (0.00)	0 (0.00)	19 (18.27)	41 (15.24)
	2021	14 (12.39)	9 (34.62)	0 (0.00)	0 (0.00)	0 (0.00)	17 (16.35)	40 (14.87)
	2022	7 (6.19)	4 (15.38)	0 (0.00)	0 (0.00)	5 (55.56)	9 (8.65)	25 (9.29)
	2023	14 (12.39)	4 (15.38)	2 (15.38)	1 (25.00)	2 (22.22)	14 (13.46)	37 (13.75)
	2024	24 (21.24)	8 (30.77)	7 (53.85)	1 (25.00)	2 (22.22)	9 (8.65)	51 (18.96)
	2025 Q1–Q2^a^	10 (8.85)	1 (3.85)	4 (30.77)	2 (50.00)	0 (0.00)	4 (3.85)	21 (7.81)
	Outcome (%)							
	Death	37 (32.74)	6 (23.08)	3 (23.08)	0 (0.00)	0 (0.00)	33 (31.73)	79 (29.37)
	Disability	3 (2.65)	0 (0.00)	0 (0.00)	0 (0.00)	0 (0.00)	0 (0.00)	3 (1.12)
	Hospitalization	40 (35.40)	15 (57.69)	2 (15.38)	1 (25.00)	5 (55.56)	22 (21.15)	85 (31.60)
	Life-threatening	11 (9.73)	0 (0.00)	0 (0.00)	0 (0.00)	3 (33.33)	16 (15.38)	30 (11.15)
	Other serious events	20 (17.70)	4 (15.38)	5 (38.46)	3 (75.00)	1 (11.11)	33 (31.73)	66 (24.54)
	Missing^b^	2 (1.77)	1 (3.85)	3 (23.08)	0 (0.00)	0 (0.00)	0 (0.00)	6 (2.23)
	Countries (%)							
	United States	59 (52.21)	9 (34.62)	8 (61.54)	0 (0.00)	5 (55.56)	83 (79.81)	164 (60.97)
	China	10 (8.85)	8 (30.77)	0 (0.00)	0 (0.00)	0 (0.00)	0 (0.00)	18 (6.69)
	Japan	6 (5.31)	3 (11.54)	0 (0.00)	0 (0.00)	0 (0.00)	0 (0.00)	9 (3.35)
	France	4 (3.54)	0 (0.00)	0 (0.00)	0 (0.00)	0 (0.00)	4 (3.85)	8 (2.97)
	Germany	7 (6.19)	0 (0.00)	0 (0.00)	0 (0.00)	0 (0.00)	1 (0.96)	8 (2.97)
	Netherlands	4 (3.54)	0 (0.00)	0 (0.00)	2 (50.00)	0 (0.00)	0 (0.00)	6 (2.23)
	Switzerland	3 (2.65)	1 (3.85)	0 (0.00)	1 (25.00)	0 (0.00)	0 (0.00)	5 (1.86)
	Spain	3 (2.65)	0 (0.00)	0 (0.00)	0 (0.00)	0 (0.00)	2 (1.92)	5 (1.86)
	Czech Republic	1 (0.88)	0 (0.00)	0 (0.00)	0 (0.00)	0 (0.00)	3 (2.88)	4 (1.49)
	United Kingdom	0 (0.00)	0 (0.00)	0 (0.00)	1 (25.00)	0 (0.00)	3 (2.88)	4 (1.49)
	Australia	3 (2.65)	0 (0.00)	0 (0.00)	0 (0.00)	0 (0.00)	1 (0.96)	4 (1.49)
	Canada	0 (0.00)	0 (0.00)	0 (0.00)	0 (0.00)	0 (0.00)	3 (2.88)	3 (1.12)
	Italy	0 (0.00)	0 (0.00)	1 (7.69)	0 (0.00)	0 (0.00)	1 (0.96)	2 (0.74)
	Brazil	2 (1.77)	0 (0.00)	0 (0.00)	0 (0.00)	0 (0.00)	0 (0.00)	2 (0.74)
	South Korea	1 (0.88)	0 (0.00)	0 (0.00)	0 (0.00)	0 (0.00)	0 (0.00)	1 (0.37)
	Belgium	1 (0.88)	0 (0.00)	0 (0.00)	0 (0.00)	0 (0.00)	0 (0.00)	1 (0.37)
	Missing^b^	9 (7.96)	5 (19.23)	4 (30.77)	0 (0.00)	4 (44.44)	3 (2.88)	25 (9.29)

a The first and second quarter of 2025.

b Missing refers to cases where data for a specific variable were not reported or could not be determined.

BCMA, B-Cell maturation antigen; IQR, Interquartile range.

Among the AE reports in the database, the great majority came from the United States (60.97%). Both generally (52.42% vs. 39.03%) and stratified by individual therapy, there were somewhat more male patients reported than female patients (e.g., axicabtagene 50.44% vs. 42.48%). With a median weight of 105.70 kg and 135.50 kg (interquartile range: 69.40–108.30 kg;106.00–165.00 kg), patients in reports connected to brexucabtagene and idecabtagene seemed to be rather overweight. The reports were mainly from two time periods: 2019–2021, as several CAR-T cell-based treatment methods were approved for marketing; In 2023–2024, the value of CAR-T therapy was recognized and actively applied in clinical practice. Over 85% of all the AE reports came from axicabtagene and tisagenlecleucel treatments; nevertheless, their age distributions clearly differed. Whereas the median age of patients with tisagenlecleucel-related AEs was 20.50 years (interquartile range: 15.00–67.50 years), the median age of those with axicabtagene-related AEs was 57.00 years (interquartile range: 47.00–65.00 years). Hospitalization is the most common outcome in reports related to axicabtagene, with 35.3% of reports. In contrast, in order of frequency, the top three results for all the data were hospitalization (31.60%), death (29.37%), and other serious events (24.54%).

#### 3.1.2 Signal result based on ROR and IC

**Figure 2:**
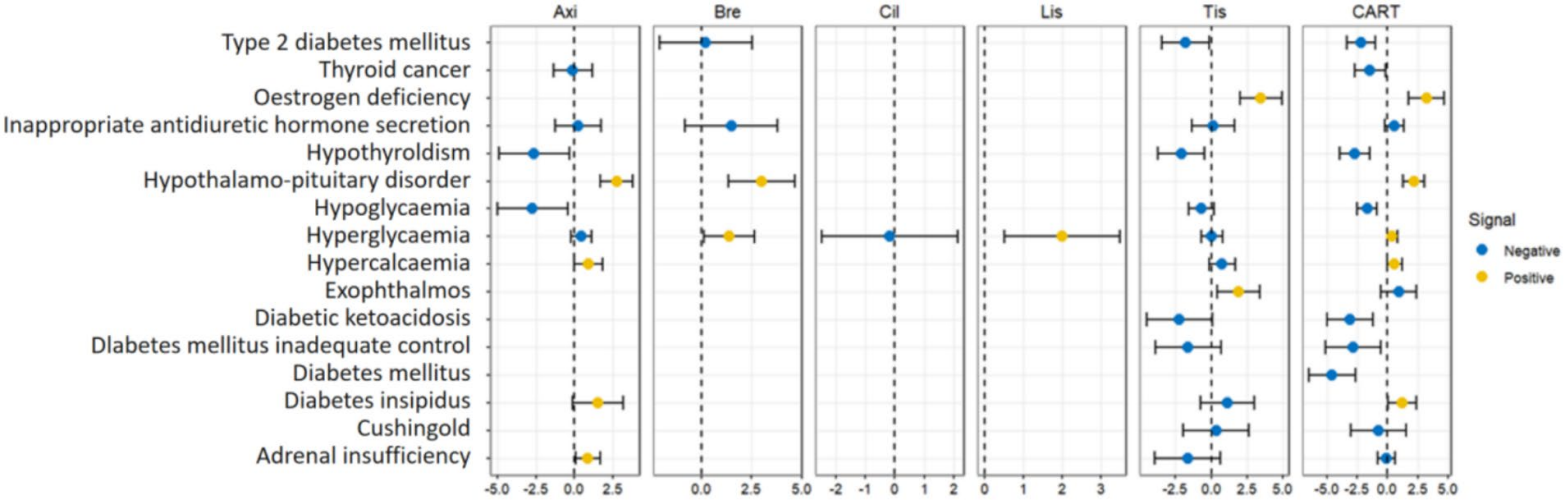
Signal detection value information component (IC) of CAR-T-associated endocrine adverse events. Data points represent point estimates, and error bars represent 95% confidence intervals. Axi, Axicabtagene; Bre, Brexucabtagene; Cil, Ciltacabtagene; Lis, Lisocabtagene; Tis, Tisagenlecleucel; CART, chimeric antigen receptor T-cell therapy.

Some trends were found in disproportional analyses based on reports of endocrine system adverse response events suspected to be associated with CART therapy in the FAERS database. We recorded a spectrum of endocrine-related side effects, arranged by frequency and severity. As shown in Supplementary Table S1, with 63 recorded cases (ROR025 = 1.03), hyperglycemia occurred the most often, followed by hypercalcemia (29 cases, ROR025 = 1.05) and adrenal insufficiency (23 cases, ROR025 = 0.62). These results suggest a possible endocrine system risk associated with CAR T-cell therapy. The remaining AEs, listed in order of frequency, included inappropriate antidiuretic hormone secretion (19 cases, ROR025 = 0.93), hypoglycaemia (18 cases, ROR025 = 0.20), hypothalamo-pituitary disorder (16 cases, ROR025 = 2.78), diabetes insipidus (9 cases, ROR025 = 1.20), type 2 diabetes mellitus (9 cases, ROR025 = 0.11), and hypothyroidism (8 cases, ROR025 = 0.08). Figure 2 and Supplementary Figure S1 help clarify the relationships of these endocrine-related AEs with CAR T-cell therapy. Besides, the low report counts for ciltacabtagene and idecabtagene, with none exceeding three reports for any AE, rendered them unsuitable for analysis using the ROR and IC methods.

Across all CAR T-cell treatments, the combined analysis revealed 14 notable AEs. Especially notable signals were hypothalamo-pituitary disorder (ROR025 = 2.78, IC025 = 1.32) and estrogen deficiency (ROR025 = 4.18, IC025 = 1.75). With respect to particular CAR T-cell therapy drugs, axicabtagene had strong signal values for hypercalcemia (ROR025 = 1.09) and hypothalamo-pituitary disorder (ROR025 = 3.70, IC025 = 1.69). In patients treated with brexucabtagene, the results also revealed statistically significant signals for hyperglycemia (ROR025 = 1.31, IC025 = 0.14) and hypothalamo-pituitary disorder (ROR025 = 3.36, IC025 = 1.36). On the other hand, tisagenlecleucel was mostly characterized by exophthalmos (ROR025 = 1.68, IC025 = 0.41) and estrogen deficiency (ROR025 = 4.92, IC025 = 2.00). Although there are many other types of adverse drug reactions in reports related to tisagenlecleucel, the remaining ones are not recognized as positive signals. Hyperglycaemia (ROR025 = 1.80, IC025 = 0.52), the sole positive signal, was also found in the adverse reaction reports related to lisocabtagene. Furthermore, owing to their rather recent approval, ciltacabtagene and idecabtagene have not yet produced enough data on endocrine system-related AEs recorded in the FAERS database, and an analysis of their possible signals has not been conducted at this point.

#### 3.1.3 Proportion of death outcomes for different endocrine system AEs

**Figure 3:**
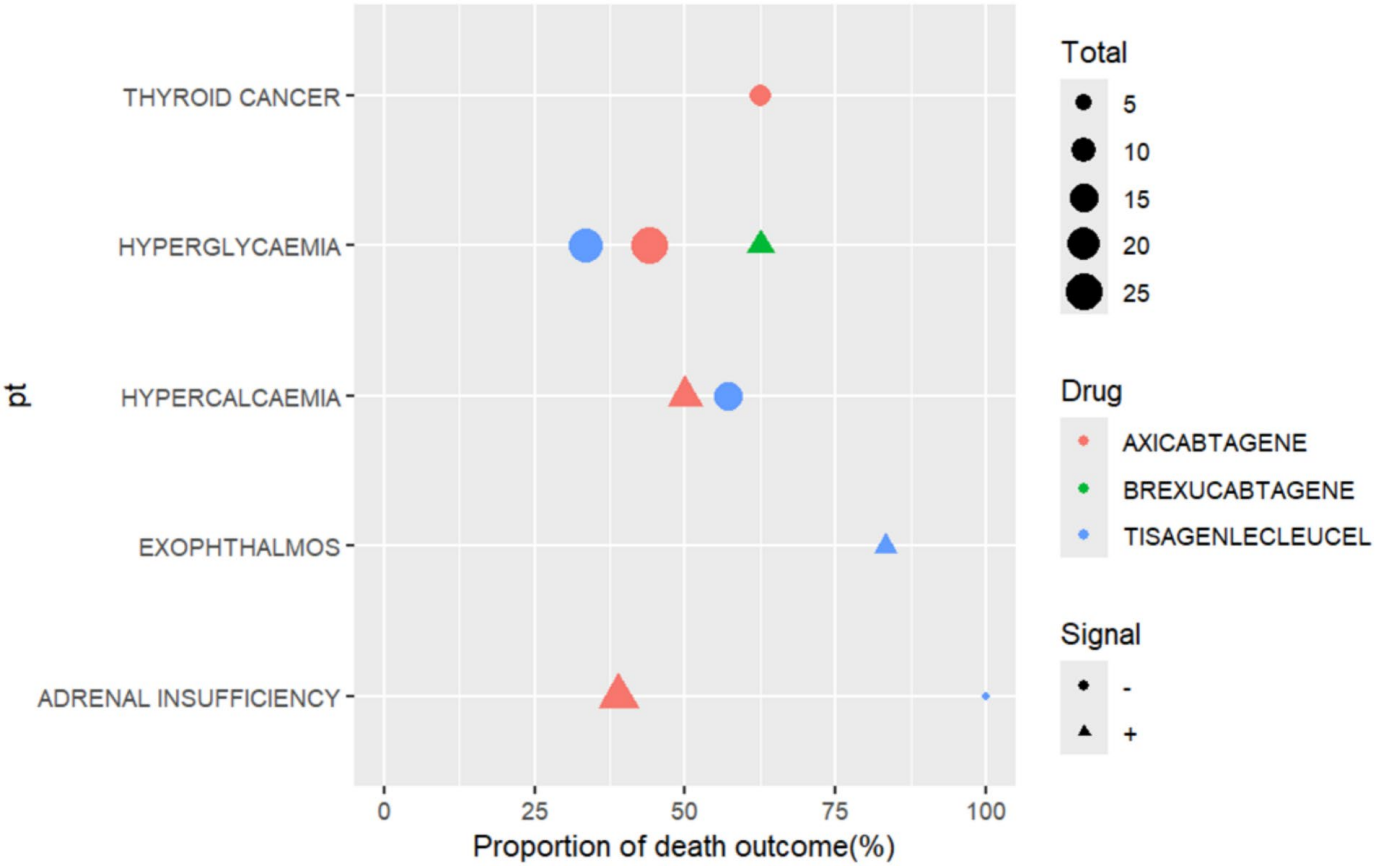
Proportion of death outcome in different endocrine system PTs. PTs, preferred terms.

Among the reports of CAR-T-related endocrine AEs, fatal outcome was documented in a subset of cases (Table 1). Evaluating AE reports depends much on the documentation and analysis of “death” outcomes since they directly affect patient safety, the assessment of the risk–benefit ratio of treatments, and the direction of clinical decision-making and regulatory policy. Thus, in this work, reports of death outcomes for particular treatments were examined via three or more recorded death inclusion criteria, as shown in Figure 3. Although the number of exophthalmos-associated reports for tisagenlecleucel treatment was rather low in the reports included in the analysis, 5 out of the 6 cases resulted in death (83.3%), hence showing the highest death rate among the positive signals detected. Hypercalcaemia-associated deaths occurred in 50.00% (7 out of 14 cases) of patients treated with axicabtagene. Although hyperglycaemia occurred in patients treated with axicabtagene, brexucabtagene and tisagenlecleucel. However, the proportion of deaths was higher in the reports of brexucabtagene, which was identified as having a positive hyperglycaemia signal (brexucabtagene 62.50% vs. axicabtagene 33.33%, tisagenlecleucel 44.00%).

#### 3.1.4 Overlap between the 4 endocrine systems AEs associated with death and CRS

**Figure 4:**
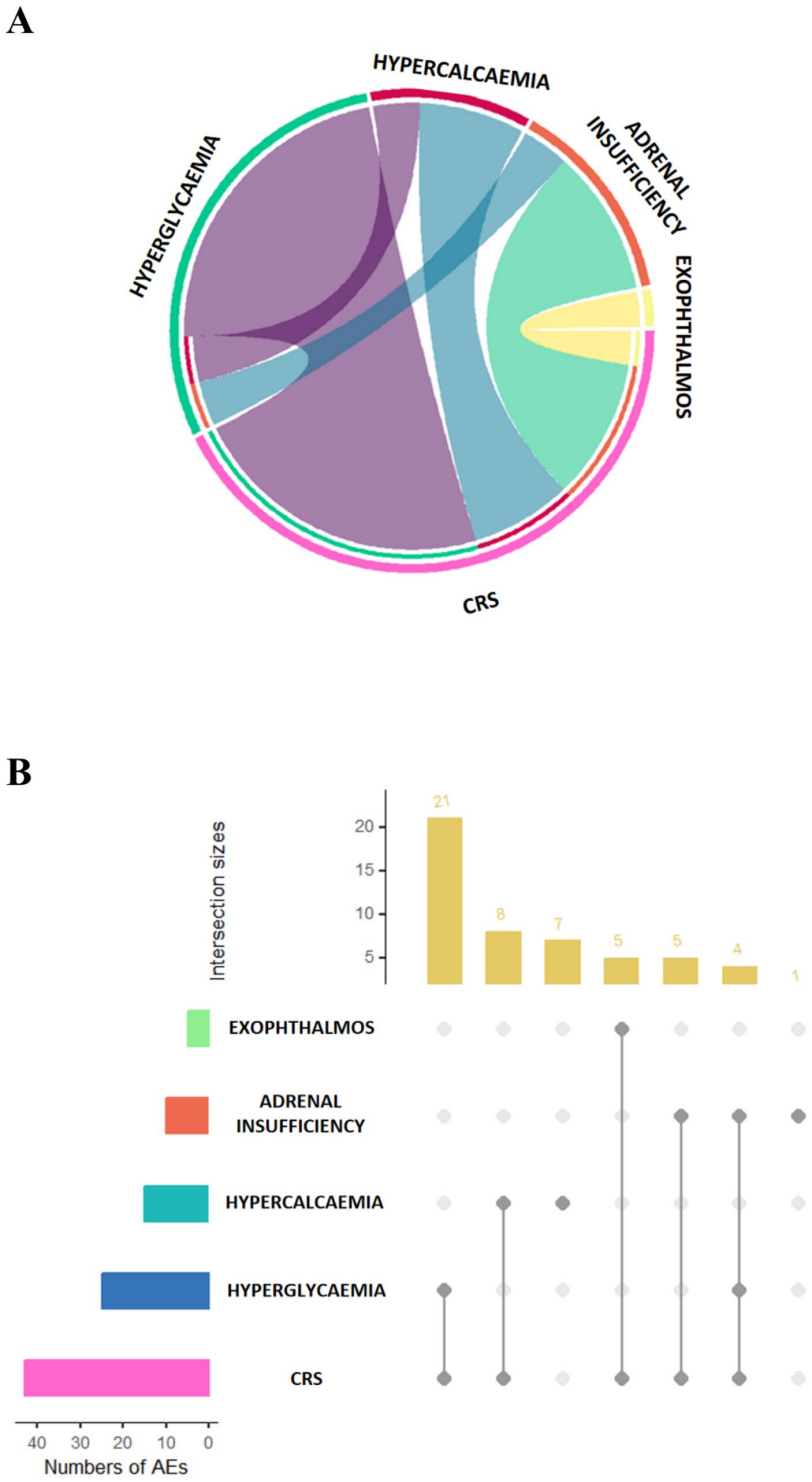
**(A)** Overlap between CRS and top 5 most frequently reported endocrine adverse events associated with death. **(B)** Upset plots of CRS and top 5 most frequently reported endocrine adverse events associated with death. CRS, cytokine release syndrome.

In our analysis, endocrine adverse events reported in conjunction with CRS were observed in several cases, with some resulting in fatal outcomes. AE reports linked to diseases that resulted in death outcomes are especially clear: exophthalmos (5 out of 6 cases, 83.33%), adrenal insufficiency (9 out of 23 cases, 39.13%), hyperglycemia (25 out of 63 cases, 39.68%), and hypercalcemia (15 out of 29 cases, 51.72%). Figure 4A shows how these four endocrine system AEs overlap with CAR T-cell therapy-related CRS. Figure 4B offers visual proof to improve the knowledge of the complexity surrounding endocrine system AEs in CAR T-cell treatment, providing further information on the particular overlap of each AE with CRS. In reports with “death” as the outcome, hyperglycemia and exophthalmos overlapped 100% with cytokine release syndrome, whereas hypercalcemia overlapped 53.8%, adrenal insufficiency overlapped 90.00%.

### 3.2 Literature search and evidence

To contextualize the pharmacovigilance signals identified through FAERS analysis, we conducted a structured and systematic literature search. The search identified a limited body of published evidence directly addressing endocrine adverse events associated with CAR T-cell therapy. After screening, only three studies met the eligibility criteria for reporting primary clinical data on specific endocrine outcomes following CAR T-cell therapy. These comprised one retrospective study addressing hyperglycemia and two case reports describing thyroid dysfunction and central diabetes insipidus, respectively (21–23).

#### 3.2.1 Hyperglycemia

The hyperglycemia signal identified in our FAERS analysis is supported by direct clinical evidence. Tienstra et al. conducted a retrospective study of 59 patients receiving CAR T-cell therapy for large B-cell lymphoma at a single center in the Netherlands between October 2017 and March 2022 (23). Among patients without pre-existing diabetes, 39% (13/33) experienced hyperglycemia (defined as glucose > 7.8 mmol/L) during hospitalization. The mean glucose concentration during hyperglycemic episodes was 13.2 ± 4.4 mmol/L, with a maximum recorded value of 27.1 mmol/L.

Notably, hyperglycemia was strongly associated with CAR T-cell therapy-related toxicities: 100% of patients who developed hyperglycemia also experienced CRS, compared with 65% of normoglycemic patients (*p* < 0.0003). Similarly, 64% of hyperglycemic patients developed immune effector cell-associated neurotoxicity syndrome (ICANS) compared to 18% in the normoglycemic group (*p* < 0.0002). Furthermore, length of hospital stay was significantly longer in patients with hyperglycemia (16 ± 7 days vs. 10 ± 3 days, *p* < 0.0001).

Importantly, while 34% of patients received dexamethasone for ICANS management, 31% of hyperglycemia cases occurred independently of corticosteroid administration, suggesting that CRS-associated inflammation—rather than iatrogenic steroid exposure alone—was a primary driver of glucose dysregulation. The authors proposed that elevated interleukin-6 (IL-6), a hallmark cytokine of CRS, directly contributes to insulin resistance and impaired glucose homeostasis through activation of JAK–STAT signaling pathways (23).

These findings provide strong clinical validation for our FAERS hyperglycemia signal and support the observed association between hyperglycemia, CRS, and adverse outcomes in the current analysis.

#### 3.2.2 Thyroid dysfunction

Although thyroid dysfunction signals were less prominent in our FAERS analysis, emerging evidence supports CAR T-cell-associated thyroid autoimmunity. Chen et al. reported two cases of Hashimoto’s thyroiditis developing after CD19-directed CAR T-cell therapy in patients with relapsed/refractory diffuse large B-cell lymphoma (21).

Both patients—a 65-year-old male and a 52-year-old female—developed elevated anti-thyroid peroxidase antibodies (TPOAb) and thyroglobulin antibodies (TgAb) following CAR T-cell infusion. Thyroid ultrasound demonstrated localized inflammation consistent with Hashimoto’s thyroiditis. Notably, thyroid function parameters (T3, T4, TSH) remained within normal limits in both cases, and thyroid antibody levels declined during follow-up. Neither patient developed clinical hypothyroidism requiring treatment.

This represents the first documented report of autoimmune thyroiditis following CAR T-cell therapy. The authors proposed that CAR T-cell infusion may disrupt thyroid immune homeostasis through mechanisms analogous to immune checkpoint inhibitor-induced thyroiditis, including disruption of T helper/regulatory T cell balance and cytokine-mediated upregulation of Fas/FasL pathways promoting thyrocyte apoptosis.

These observations suggest that subclinical thyroid autoimmunity may be underrecognized in CAR T-cell recipients and warrant prospective monitoring.

#### 3.2.3 Hypothalamic–pituitary dysfunction and central diabetes insipidus

Our FAERS analysis identified hypothalamo-pituitary disorder as a significant signal, particularly associated with axicabtagene ciloleucel (ROR₀₂₅ = 3.70, IC₀₂₅ = 1.69). A case report by Koch et al. provides direct clinical evidence supporting this finding (22).

The authors described a patient with relapsed MYC/BCL-6 rearranged high-grade B-cell lymphoma with CNS involvement who received tisagenlecleucel and subsequently developed central diabetes insipidus (cDI) along with a Guillain-Barré-like syndrome. Central diabetes insipidus manifested on day +11 post-infusion with polyuria up to 950 mL/h, markedly decreased urine sodium concentration and osmolality, and rapid response to desmopressin treatment. Serial MRI examinations revealed no structural abnormalities of the pituitary gland or hypothalamus, and other pituitary hormones (TSH, LH, FSH, prolactin, growth hormone) remained normal.

Cerebrospinal fluid (CSF) analysis demonstrated CAR T-cell enrichment, with CAR⁺ cells constituting 37% of a mild lymphocytic pleocytosis—more than 2-fold higher than the proportion in peripheral blood—suggesting disproportionate CAR T-cell expansion within the CNS. The cDI resolved over several weeks with desmopressin treatment and was successfully tapered before hospital discharge on day +56.

The authors hypothesized that structures outside the blood–brain barrier, such as the pituitary gland, may be more accessible to CAR T-cells and therefore susceptible to immune-mediated inflammation affecting hormone homeostasis. While overt hypophysitis was ruled out radiologically, a direct or indirect role of CAR T-cells in disrupting antidiuretic hormone production remains plausible.

This case represents the first documented report of central diabetes insipidus following CAR T-cell therapy and provides clinical validation for the hypothalamic–pituitary dysfunction signal identified in our FAERS analysis.

## 4 Discussion

### 4.1 Overview of endocrine safety profile in CAR T-cell therapy

The aim of this study is to retrospectively perform an disproportionality analysis of reports of endocrine system adverse response events in the FAERS database that are suspected to be associated with CART therapy to investigate the potential endocrine system toxicity of CART therapy. Although endocrine system AEs show some therapeutic specificity, studies on AEs linked to CAR T-cell therapy have focused mostly on the neurological and hematological systems, together with cytokine release syndrome. As such, information on endocrine system AEs has not yet been sufficiently addressed (24, 25). The disproportionate reporting of endocrine-related AEs in patients receiving CAR-T therapy suggests some potential safety signals that warrants further investigation. Given the increasing frequency of CAR T-cell therapy, it is essential to evaluate how it might affect the endocrine system.

### 4.2 Gender disparities in reported AEs

The number of endocrine system adverse events reported in mostCAR-T cell therapy products, including ascibutane, tisagrelu, brexabutane, and risocabutane, was higher in men than in women (Table 1). Epidemiological data indicate that hematological malignancies impose a greater burden on males (2), with leukemia incidence showing a male-to-female ratio of 1.4 (26). Male sex is also an established risk factor for survival across various hematological malignancies in both adult and pediatric populations (27). In present studies, we noted that endocrine system AEs occurred somewhat more frequently in male with hematologic tumors receiving CAR T-cell therapy than in their female counterparts. Gender variations in endocrine AEs have not been fully investigated in previous studies examining AEs linked with CAR T-cell therapy since most of them have focused on cytokine release syndrome (5), hematological effects (28), and the central nervous system. Consequently, gender disparities in endocrine AEs need to be thoroughly investigated.

### 4.3 Signal detection: metabolic and hypothalamic–pituitary disorders

The AE most frequently mentioned in reports of endocrine system adverse response events suspected to be associated with CAR T cell therapy were hyperglycemia (63 cases, 23.42%), hypercalcemia (29 cases, 10.78%), adrenal insufficiency (23 cases, 8.55%), inappropriate secretion of antidiuretic hormone (19 cases, 7.06%), and hypothalamo-pituitary disorder (16 cases, 5.95%). The report claims that hyperglycemia in 19% of cases constituted any grade of treatment-emergent AE (29). The product information does not cover the possibility of negative events linked with hypothalamo-pituitary disease, exophthalmos, and estrogen deficiency following the use of tisagenlecleucel (30, 31). Furthermore, negative responses associated with hyperglycemia were recorded for Tecartus in the ZUMA-2 and ZUMA-3 studies and were categorized as “VERY COMMON” (≥ 1/10) (32–34). Since the product information for CAR T-cell therapy lists only hyperglycemia and hypercalcemia under the category of metabolic and nutritional disorders, overall, the information about AEs in the endocrine system is mostly silent (34). From the perspective of the endocrine system, however, we discovered that CAR T-cell therapy might also cause side effects, including exophthalmos, estrogen deficiency, and hypothalamo-pituitary disorders. Given the need for education and training for clinical practitioners, our study highlights the potential relationship between CAR T-cell therapy and endocrine system toxicity. During CAR T-cell therapy, we advise routinely evaluating patients for endocrine function to guarantee patient safety. Thyroid function tests, adrenocorticotropic hormone level measurements, and blood glucose monitoring should all be part of this assessment—but not the only ones.

The three main disproportionate signals linked with axicabtagene were hypercalcemia (ROR025 = 1.09), adrenal insufficiency (ROR025 = 1.16, IC025 = 0.07), hypothalamo-pituitary disorder (ROR025 = 3.70, IC025 = 1.69), and diabetes insipidus (ROR025 = 1.20). Hypercalcemia, defined as serum calcium >10.5 mg/dL (2.5 mmol/L), is associated with hyperthyroidism or malignancy in approximately 90% of cases (35). Moderate-to-severe hypercalcemia (12.0–13.9 mg/dL) can cause altered mental status or coma, necessitating prompt intervention including intravenous hydration and bisphosphonates (36). Adrenal insufficiency, characterized by inadequate cortisol production (37), can precipitate life-threatening adrenal crisis if unrecognized (38). These endocrine disturbances, while rare, carry significant clinical implications. Our disproportionality analysis identified several specific safety signals across different CAR-T products. Both axicabtagene and brexucabtagene showed positive signals for hypothalamo-pituitary disorder (ROR025 = 3.36, IC025 = 1.36), representing the strongest endocrine signals observed. Given the hypothalamic–pituitary axis’s central role in endocrine regulation, dysfunction at this level carries serious implications for patient development and survival. Clinical evaluation of such disorders requires comprehensive assessment of both endocrine function and pituitary anatomy, with treatment tailored to the specific presentation (39). For brexucabtagene, hyperglycemia also emerged as a significant signal (ROR025 = 1.31, IC025 = 0.14). Previous studies suggest this may be mediated by cytokine release syndrome (40), particularly IL-6 elevation, which is associated with insulin resistance (23). This mechanistic link underscores the potential for CAR-T therapy to contribute to hyperglycemia development. Tisagenlecleucel demonstrated two distinct positive signals: exophthalmos (ROR025 = 1.68, IC025 = 0.41) and estrogen deficiency (ROR025 = 4.92, IC025 = 2.00). Exophthalmos, primarily associated with thyroid dysfunction, accounts for approximately 90% of bilateral cases (41) and can lead to serious ocular complications including corneal dryness, infection risk, ulceration, and visual impairment if severe (42). While no mechanistic studies have specifically addressed tisagenlecleucel-induced estrogen deficiency, this signal warrants clinical attention.

Synthesizing these findings, we hypothesize that CAR-T therapy may disrupt hypothalamic–pituitary-glandular axis function, manifesting as multifaceted endocrine disturbances. Our analysis reveals that CAR-T-related endocrine toxicity encompasses not only biochemical abnormalities (glucose/calcium dysregulation) but also functional impairment of multiple endocrine glands, including pituitary, thyroid, adrenal, and gonadal involvement. This broad spectrum of potential toxicity necessitates vigilant monitoring and proactive management of endocrine AEs in patients receiving CAR-T therapy. Notably, mortality associated with these endocrine events was substantial: 83.33% in patients with tisagenlecleucel-induced exophthalmos, and 62.50% in those with brexucabtagene-associated hyperglycemia. These striking figures underscore the critical importance of early recognition and prompt intervention. Clinicians should maintain a high index of suspicion for endocrine toxicity when administering CAR-T therapies, as timely action may mitigate patient risk and improve overall treatment safety.

### 4.4 The potential interplay between CRS, endocrine toxicity, and mortality

Over the past 10 years, CAR T-cell therapy has made major progress in the treatment of hematologic cancers. However, its development is still limited by concurrent cytokine release syndrome (43, 44). While no studies have yet documented the interaction between concomitant CRS and endocrine toxicity linked with CAR T-cell therapy, based on our analysis of the FAERS database, we recommend more comprehensive monitoring of patients with both CRS and endocrine AE after receiving CART therapy. When CRS co-occurrence was considered, the proportion of fatal outcomes was higher among patients with hyperglycemia (25/25, 100%) and exophthalmos (5/5, 100%), corresponding to 39.68% (25/63) of hyperglycemia cases and 83.33% (5/6) of exophthalmos cases, respectively (Figure 4). However, due to the small number of cases, these findings are statistically unstable and may reflect reporting bias, as severe or fatal cases are more likely to be reported to FAERS. Additionally, it is important to acknowledge that CRS itself is a known potentially fatal complication of CAR-T therapy; therefore, the contribution of endocrine dysfunction to mortality in these overlapping cases cannot be definitively established. Given the limited number of reports and the inherent biases of spontaneous reporting databases, we emphasize that these signals represent potential associations rather than established causal relationships. The complexity of cytokine release syndrome—which may negatively impact outcomes in patients with concurrent hyperglycemia, adrenal insufficiency, and exophthalmos—warrants further investigation. We recommend that researchers and clinicians remain attentive to these possible endocrine complications, while recognizing that prospective studies are needed to confirm these preliminary observations. These three negative effects have an anatomical basis in common even if they might not seem directly related. Hyperglycemia is usually related to either insufficient insulin production or insulin resistance. The inability of the adrenal glands to secrete hormones correctly results in an imbalance in the body’s hormone levels, indicating adrenal insufficiency. Furthermore, the development of proptosis is sometimes connected to hyperthyroidism. One can classify the relevant glands—pancreas, adrenal glands, and thyroid—as elements of the hypothalamic–pituitary–glandular axis system. Given the positive signals of hypothalamo-pituitary dysfunction observed in several products listed in the previous section, along with the systemic inflammatory response and organ dysfunction resulting from excessive release of various cytokines (such as IL-6 and TNF-α) during cytokine release syndrome, CRS may indirectly aggravate disorders of the endocrine system. This aggravation might present as changes in ocular tissues, adrenal activity, and blood glucose levels. Given the exploratory nature of this pharmacovigilance analysis and the inherent limitations of spontaneous reporting data, these mortality findings should be interpreted with caution. While the observed frequency of fatal outcomes among patients with concurrent endocrine AEs and CRS raises a hypothesis worthy of further investigation, we suggest that clinicians maintain awareness of this potential association and consider close monitoring of endocrine function in patients receiving CAR-T therapy.

### 4.5 Integration of pharmacovigilance signals with published evidence

The structured literature review identified three clinical studies directly addressing endocrine AEs following CAR-T therapy, providing validation and context for our FAERS signals.

For hyperglycemia, Tienstra et al. reported a 39% incidence in non-diabetic patients, with strong associations between hyperglycemia and both CRS and ICANS (23). This validates our FAERS finding of hyperglycemia as the most frequently reported endocrine AE. The mechanistic link between IL-6-mediated inflammation during CRS and insulin resistance provides biological plausibility, while the association with prolonged hospitalization underscores clinical significance.

For thyroid dysfunction, Chen et al. documented Hashimoto’s thyroiditis in two patients following CD19 CAR-T therapy—the first published evidence of autoimmune thyroid disease in this setting (21). Although our FAERS signal for thyroid dysfunction was relatively weak, this case series suggests subclinical thyroid autoimmunity may be underrecognized, particularly as both patients maintained normal thyroid function. The proposed mechanism involving disrupted T cell homeostasis parallels pathways in immune checkpoint inhibitor-associated thyroiditis.

For hypothalamic–pituitary dysfunction, Koch et al. described central diabetes insipidus following tisagenlecleucel, providing direct clinical validation for our FAERS signal (22). CAR-T cell enrichment in CSF suggests potential CNS-directed immune activity extending beyond classic ICANS manifestations. Notably, this case demonstrates that endocrine complications can occur independently of, and with different temporal patterns than, ICANS, highlighting the need for systematic endocrine surveillance beyond standard neurotoxicity monitoring.

Despite these observations, the published literature on CAR-T-associated endocrine AEs remains remarkably limited. For several signals identified in our analysis—including adrenal insufficiency, hypercalcemia, and gonadal axis dysfunction—no direct CAR-T clinical evidence was identified, necessitating reliance on mechanistic inference and ICI-derived analogous data. This evidence gap underscores the hypothesis-generating nature of our pharmacovigilance findings and highlights an underexplored area in CAR-T safety research warranting prospective investigation.

### 4.6 Clinical implications and recommendations

CAR T-cell therapy unquestionably holds promise for treating hematologic malignancies. However, its unique toxicity profile—particularly cytokine release syndrome—remains a major concern for patients and clinicians, with potentially fatal outcomes (45). International organizations have begun addressing these challenges: the European Myeloma Network recommends premedication and frequent CRS assessment (46), while NCCN guidelines emphasize close monitoring before, during, and after infusion to enable early recognition and intervention for treatment-related adverse events (47). Despite this growing attention to CAR-T toxicity, few systematic studies have investigated the potential for significant endocrine system adverse events. Our findings address this gap, demonstrating that CAR-T therapy maybe affect multiple levels of the hypothalamic–pituitary-glandular axis, manifesting as glucose dysregulation, thyroid dysfunction, adrenal insufficiency, and gonadal axis disturbances.

From a clinical perspective, these findings suggest that baseline and early post-infusion assessment of glucose metabolism and pituitary-related symptoms may be clinically valuable, particularly in patients experiencing CRS. Given the often nonspecific presentation of endocrine disturbances, heightened clinical vigilance could facilitate earlier recognition and supportive management. We therefore recommend systematic monitoring of endocrine function in patients receiving CAR-T therapy to enable timely identification and intervention, potentially preventing serious complications and improving overall treatment safety.

### 4.7 Limitations

Several limitations warrant consideration. First, the FAERS database has inherent methodological limitations, including potential data duplication, incomplete information, reporting bias, and the absence of denominator data precluding true incidence rate calculations (48, 49). Consequently, the identified signals should be interpreted as hypothesis-generating rather than confirmatory of causal relationships. Second, as a retrospective pharmacovigilance analysis, this study lacked access to individual patient-level clinical data. The observed endocrine signals may be influenced by concomitant medications (particularly corticosteroids), underlying malignancy-related effects, or lymphodepleting conditioning regimens, and these confounding factors cannot be fully disentangled from direct CAR T-cell effects. Third, the literature component was designed as systematic evidence mapping rather than a formal systematic review; the limited number of directly relevant publications (*n* = 3) precluded quantitative synthesis. However, this evidence gap itself represents a significant finding. Finally, due to low report counts, ciltacabtagene autoleucel and idecabtagene vicleucel could not be included in disproportionality analysis.

Despite these limitations, our integrated analysis provides a large-scale real-world assessment of potential endocrine safety signals associated with CAR T-cell therapy and offers actionable insights for clinical practice and future research.

## 5 Conclusion

CAR T-cell therapy represents a transformative treatment paradigm for advanced hematologic malignancies, yet its potential endocrine toxicity profile remains under-recognized and inadequately characterized. This integrated pharmacovigilance analysis, combining FAERS-based disproportionality analysis with systematic evidence mapping, identifies a spectrum of endocrine adverse event signals that warrant heightened clinical attention.

Hyperglycemia emerged as the most frequently reported signal, with direct clinical evidence supporting its association with cytokine release syndrome and adverse hospitalization outcomes. Hypothalamic–pituitary disorders, including central diabetes insipidus, and adrenal insufficiency represent lower-frequency but clinically significant signals with potential for life-threatening complications if unrecognized. Estrogen deficiency associated with tisagenlecleucel demonstrated the strongest signal strength, highlighting potential gonadal toxicity warranting attention in reproductive-age patients. The systematic literature mapping further revealed that most identified signals lack direct prospective validation, emphasizing both the novelty of these findings and the critical need for further investigation.

Notably, the co-occurrence of endocrine adverse events with cytokine release syndrome was associated with markedly elevated mortality rates, suggesting that endocrine emergencies may be masked by or attributed to CRS manifestations, potentially contributing to delayed recognition and adverse outcomes.

Given the vital physiological role of the endocrine system and the severe clinical consequences of these adverse events, current safety monitoring protocols may be insufficient. We underscore the necessity for prospective studies to establish true incidence rates and risk factors, mechanistic investigations into CAR T-cell-induced endocrine dysregulation, and the integration of systematic endocrine monitoring into routine post-infusion care. Implementation of structured baseline and longitudinal endocrine assessment may facilitate early detection and management of these complications, potentially reducing preventable morbidity and mortality in CAR T-cell recipients.

## Data Availability

The original contributions presented in the study are included in the article/Supplementary material, further inquiries can be directed to the corresponding authors.
