# Supplementary material for: Spectrum and prognosis of CAR-T-related endocrine toxicity: glucose/calcium dysregulation, pituitary axis disorders, and overlap with CRS
Source: Front Med (Lausanne). 2026 Apr 14;13:1786129. doi: 10.3389/fmed.2026.1786129 (PMC13121071; doi:10.3389/fmed.2026.1786129)
Supplement: Supplementary file 1 [file Supplementary_File_1.docx]

**Supplementary Material**


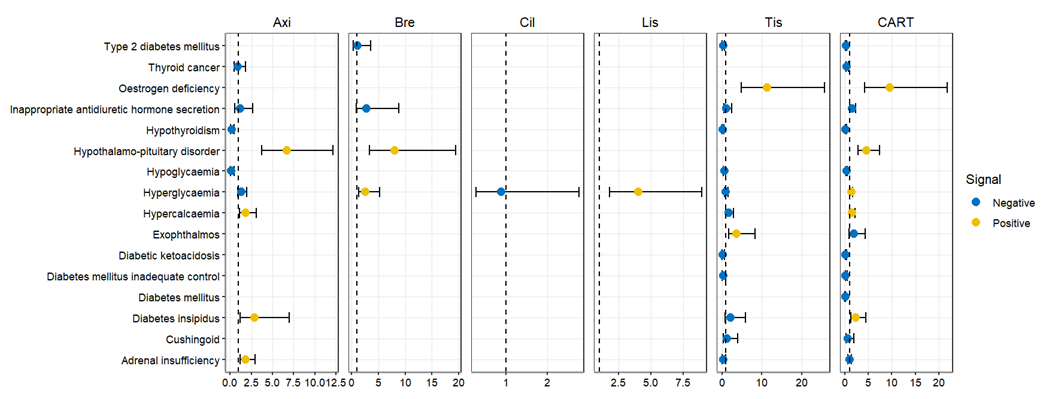


**Figure S1 Signal detection value reporting odds ratio (ROR) of CAR-T-associated endocrine adverse events**

Abbreviations: Axi, Axicabtagene; Bre, Brexucabtagene; Cil, Plantagenet; Lis, Lisocabtagene; Tis, Tisagenlecleucel; CART, chimeric antigen receptor T-cell therapy.

**Table S1 Signal detection of CAR-T associated PTs in endocrine system**

| **PTs** | **Axi** | | | **Bre** | | | **Lis** | | | **Tis** | | | **CAR-T** | | |
| --- | --- | --- | --- | --- | --- | --- | --- | --- | --- | --- | --- | --- | --- | --- | --- |
|  | **a** | **ROR_025_** | **IC_025_** | **a** | **ROR_025_** | **IC_025_** | **a** | **ROR_025_** | **IC_025_** | **a** | **ROR_025_** | **IC_025_** | **a** | **ROR_025_** | **IC_025_** |
| Hyperglycaemia | 25 | 0.91 | -0.25 | 8 | **1.31** | **0.14** | 6 | **1.80** | **0.52** | 21 | 0.67 | -0.70 | 63 | **1.03** | -0.02 |
| Hypercalcaemia | 14 | **1.09** | -0.04 |  |  |  |  |  |  | 14 | 1.00 | -0.17 | 29 | **1.05** | -0.03 |
| Adrenal insufficiency | 18 | **1.16** | **0.07** |  |  |  |  |  |  | 3 | 0.10 | -3.95 | 23 | 0.62 | -0.81 |
| Inappropriate antidiuretic hormone secretion | 6 | 0.53 | -1.24 | 3 | 0.91 | -0.79 |  |  |  | 6 | 0.49 | -1.36 | 19 | 0.93 | -0.23 |
| Hypoglycaemia | 3 | 0.05 | -5.01 |  |  |  |  |  |  | 15 | 0.37 | -1.60 | 18 | 0.20 | -2.47 |
| Hypothalamo-pituitary disorder | 11 | **3.70** | **1.69** | 5 | **3.36** | **1.36** |  |  |  |  |  |  | 16 | **2.78** | **1.32** |
| Diabetes insipidus | 5 | **1.20** | -0.13 |  |  |  |  |  |  | 4 | 0.82 | -0.77 | 9 | **1.20** | **0.03** |
| Type 2 diabetes mellitus |  |  |  | 3 | 0.38 | -2.06 |  |  |  | 5 | 0.12 | -3.44 | 9 | 0.11 | -3.37 |
| Hypothyroidism | 3 | 0.05 | -4.91 |  |  |  |  |  |  | 5 | 0.10 | -3.76 | 8 | 0.08 | -3.94 |
| Thyroid cancer | 8 | 0.46 | -1.37 |  |  |  |  |  |  |  |  |  | 8 | 0.18 | -2.69 |
| Exophthalmos |  |  |  |  |  |  |  |  |  | 6 | **1.68** | **0.41** | 6 | 0.86 | -0.55 |
| Oestrogen deficiency |  |  |  |  |  |  |  |  |  | 6 | **4.92** | **2.00** | 6 | **4.18** | **1.75** |
| Diabetes mellitus |  |  |  |  |  |  |  |  |  |  |  |  | 4 | 0.02 | -6.46 |
| Diabetic ketoacidosis |  |  |  |  |  |  |  |  |  | 3 | 0.07 | -4.51 | 4 | 0.04 | -4.96 |
| Cushingoid |  |  |  |  |  |  |  |  |  | 3 | 0.41 | -1.95 | 3 | 0.19 | -3.02 |
| Diabetes mellitus inadequate control |  |  |  |  |  |  |  |  |  | 3 | 0.10 | -3.91 | 3 | 0.05 | -5.10 |

Abbreviations: PTs, preferred terms; Axi, Axicabtagene; Bre, Brexucabtagene; Cil, Ciltacabtagene; Lis, Lisocabtagene; Tis, Tisagenlecleucel; CART, chimeric antigen receptor T-cell therapy.
